# A systematic review on sex differences in adverse drug reactions related to psychotropic, cardiovascular, and analgesic medications

**DOI:** 10.3389/fphar.2023.1096366

**Published:** 2023-05-02

**Authors:** Yuting Shan, Lee Cheung, Yuqi Zhou, Yingbo Huang, R. Stephanie Huang

**Affiliations:** Department of Experimental and Clinical Pharmacology, College of Pharmacy, University of Minnesota, Minneapolis, MN, United States

**Keywords:** sex differences, adverse drug reactions, psychotropic, cardiovascular, analgesic

## Abstract

**Background and objective:** Adverse drug reactions (ADRs) are the main safety concerns of clinically used medications. Accumulating evidence has shown that ADRs can affect men and women differently, which suggests sex as a biological predictor in the risk of ADRs. This review aims to summarize the current state of knowledge on sex differences in ADRs with the focus on the commonly used psychotropic, cardiovascular, and analgesic medications, and to aid clinical decision making and future mechanistic investigations on this topic.

**Methods:** PubMed search was performed with combinations of the following terms: over 1,800 drugs of interests, sex difference (and its related terms), and side effects (and its related terms), which yielded over 400 unique articles. Articles related to psychotropic, cardiovascular, and analgesic medications were included in the subsequent full-text review. Characteristics and the main findings (male-biased, female-biased, or not sex biased ADRs) of each included article were collected, and the results were summarized by drug class and/or individual drug.

**Results:** Twenty-six articles studying sex differences in ADRs of six psychotropic medications, ten cardiovascular medications, and one analgesic medication were included in this review. The main findings of these articles suggested that more than half of the ADRs being evaluated showed sex difference pattern in occurrence rate. For instance, lithium was found to cause more thyroid dysfunction in women, and amisulpride induced prolactin increase was more pronounced in women than in men. Some serious ADRs were also found to exert sex difference pattern, such as clozapine induced neutropenia was more prevalent in women whereas simvastatin/atorvastatin-related abnormal liver functions were more pronounced in men.

## 1 Introduction

Adverse drug reactions (ADRs), or drug side effects, are defined as harmful, unintended events resulting from the use of medications. For a new drug entity to be approved by the US Food and Drug Administration (FDA), its safety and potential ADRs must be assessed during the investigational stage. According to a recent study, about 17% of the investigational drugs failed in phase 3 or pivotal trials because of safety concerns (Hwang et al., 2016). Even for the drugs that have been approved for clinical use, their ADRs can still be concerning. Serious ADRs were shown to result in over 100,000 deaths per year, making it the fourth leading cause of death in the US (Giacomini et al., 2007). Other less severe ADRs have been associated with drug discontinuation, poor adherence, and suboptimal treatment outcomes (DiBonaventura et al., 2012). Therefore, it is of great translational value to identify the risk factors for common or serious ADRs, so that clinical monitoring or medication change can be applied accordingly.

As an easy-to-use patient characteristic, sex has been identified as an important predictor in both disease incidence and treatment outcomes. For instance, among non-smokers, women are found to have higher risk of developing lung cancer compared to men (Ragavan and Patel, 2022), whereas women tend to respond better to epidermal growth factor receptor (EGFR) inhibitors, a targeted therapy for lung cancer, than men (Chen et al., 2013). Likewise, the role of sex in the likelihood of ADRs has been evaluated in numerous medications. One illustrative example is zolpidem, a medication used to treat insomnia. Twenty years after its approval to the market, FDA issued Drug Safety Communication (U.S. Food and Drug Administration, 2022) to require a decreased initial dose of zolpidem in women, due to the accumulating evidence indicating that women experience more driving impairment than men under the same recommended dose (Verster and Roth, 2012; Farkas et al., 2013). Subsequent pharmacokinetic studies found that the same dose resulted in significantly higher zolpidem plasma concentration in women than in men (Olubodun et al., 2003; Greenblatt et al., 2014; Greenblatt et al., 2022), which might be able to explain the higher incidence of zolpidem-related ADRs in women. Even though sex difference has gained increasing awareness nowadays, many of the existing clinical trials did not provide sex specific data when evaluating drug efficacy and safety (Hayes and Redberg, 2008; Beery and Zucker, 2011), making it challenging to promote sex-aware prescribing for most of the medications.

Here, we systematically review and summarize the existing literature evaluating sex differences in ADRs to address the fundamental question that whether sex should be considered in drug prescription to prevent/minimize ADRs. If so, for which drugs/drug classes. To summarize and discuss the findings of the included literature, we classified the medications into their therapeutic area. We chose to focus on psychotropic, cardiovascular, and analgesic medications because the above three drug classes are the top categories with sex difference studies available from our web scraping results. Furthermore, the above three drug categories yield the largest number of the “most prescribed drugs” in the US (Fuentes et al., 2018), supporting their broad use and clinical impact. It is to note that oncology mediations were not evaluated in this review due to the inherent cytotoxic effects and the different standard in the ADR recordings (Nguyen et al., 2019). By summarizing the main findings of the commonly used medications in the three drug classes, we aim to facilitate clinical decision making by improving the current understanding of sex differences in ADRs. More importantly, this review highlights the need of further research on sex-aware evaluation of ADRs.

## 2 Materials and methods

### 2.1 Search strategy

To search for evidence of sex difference in ADRs, we performed web scraping in PubMed using a R package “easyPubMed” (Fantini, 2019). The keywords used for searching were drugs of interests, sex difference (and its related terms), and side effects (and its related terms). The full list of searching terms and other restrictions can be found in Supplementary Table S1. For the drugs of interests, we used a list of 1,819 drugs which have established human targets and the corresponding ADRs recorded in clinical trials from a previously published paper by (Nguyen et al., 2019). Web scraping was performed in March 2022.

### 2.2 Study selection

All studies resulted from web scraping were considered regardless of study design or date of publication. We first performed an initial screening on the title and abstract to exclude unrelated literature. Then, we did full-text review with the focus on psychotropic, cardiovascular, and analgesic medications. To ensure the drugs included in the review belong to the above three categories, we used Anatomical Therapeutic Chemical (ATC) Classification developed by WHO, (2023) as the reference. Studies were excluded during the full-text review if 1. language was not English; 2. the drug of interest was not in the 1,819 drug list; 3. sex difference was evaluated in drug efficacy rather than ADRs; 4. sex difference in ADRs were caused by a combination of drugs rather than a specific drug; 5. significance level was not reported; 6. The ADR being evaluated is not a well-established ADR as endorsed by Micromedex (IBM MICROMEDEX, 2022). Review articles were also inspected to identify additional original studies to be included.

### 2.3 Data collection

For each of the study included in this review, the following information was collected: 1. study design; 2. race and age (adults or children) of the study population; 3. health status of the participants (healthy volunteers or patients with specific diseases); 4. number of male and female participantsin the study; 5. drug of interest; 6. dosing regimen; 7. ADRs being inspected in the study; 8. results for sex difference study in ADRs (male-biased ADR, female-biased ADR, or ADR with no sex difference); 9. any pharmacokinetic (PK) measurement if applicable.

## 3 Results

### 3.1 Study characteristics

**FIGURE 1:**
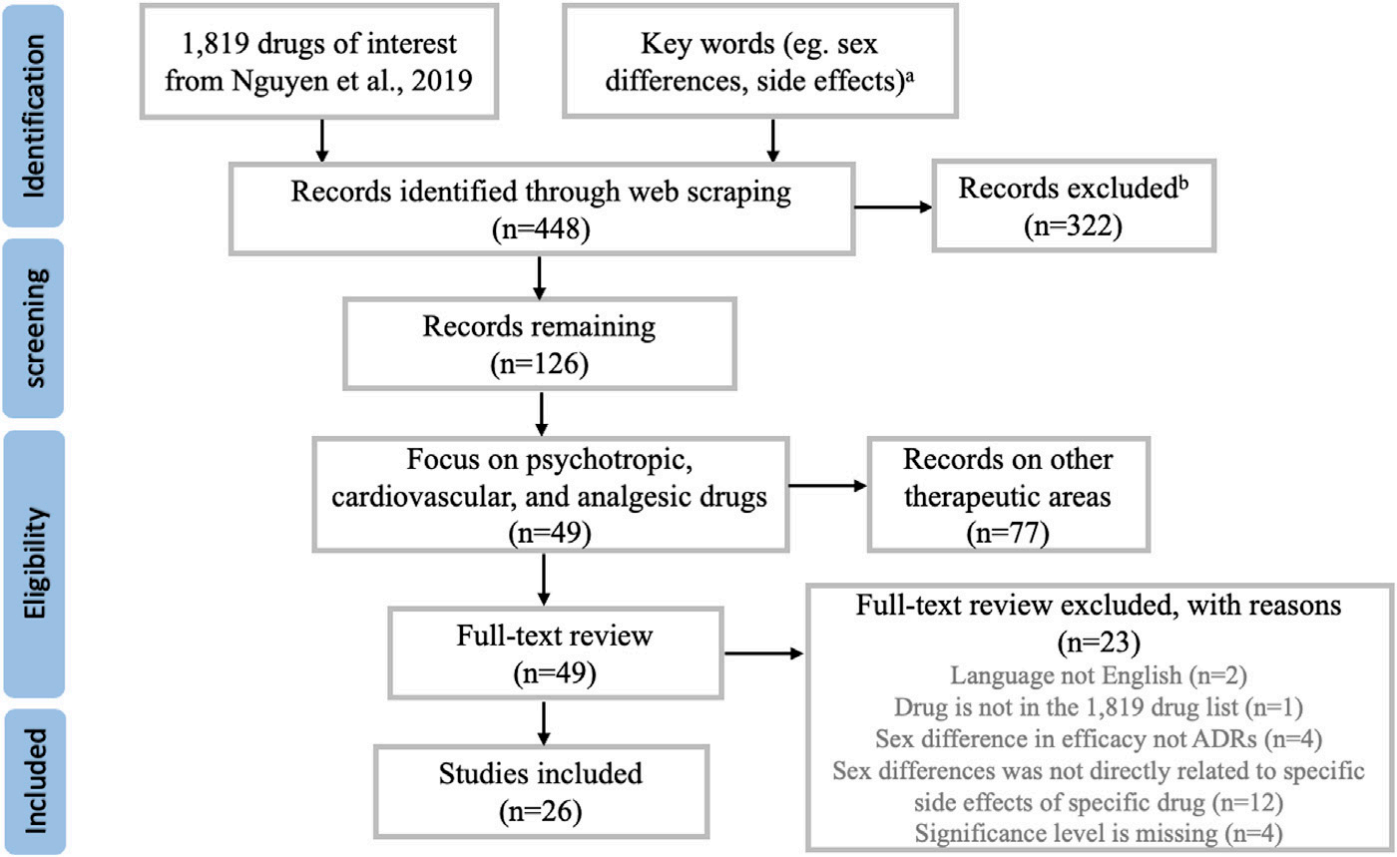
Overview of the study selection process. Note (A) A complete list of the keywords used in web scraping can be found in Supplementary Table S1. Note (B) Most studies being excluded here were not related to drug side effects, or not related to human subjects.

Literature search for the 1,819 drugs through web scraping retrieved 448 unique publications. Figure 1 summarized the process of study selection, which resulted in a total of 26 studies included in this review. The characteristics of each study such as drug of interest, study design, number of subjects, dosing regimen, etc. were recorded in Table 1. Sex differences in ADRs were summarized for six psychotropic medications, ten cardiovascular medications, and one analgesic medication. The rest of the result session was structured to first briefly introduce the clinical significance and common ADRs of the medications, followed by the evidence of sex difference in common or serious ADRs related to the drug of interest.

**TABLE 1:** Characteristics of studies included.

*Studies*	*Drugs*	*Study design*	*Number of subjects (Male/Female)*	*Dosing regimen*	*Race*	*Children/Adults*	*Patients/volunteers*	*PK measurement*
Psychotropic medications								
Özerdem et al. (2014)	lithium	Retrospective naturalistic study	240 (104/136)	Individualized dosing	NR	adults	bipolar disorder patients	Serum lithium level was higher in women
Henry (2002)	lithium	Retrospective study	60 (22/38)	Individualized dosing	NR	adults	Type I bipolar disorder patients	NR
Öhlund et al. (2018)	lithium	Retrospective cohort study	423 (185/238)	Individualized dosing	Swedish	adults	bipolar disorder patients	NR
Hoekstra et al. (2021)	amisulpride	Prospective randomized study	144 (93/51)	aripiprazole 15 mg/day	Predominantly Caucasian	adults	schizophrenia patients	amisulpride and aripiprazole level were higher in women
	aripiprazole			olanzapine 10 mg/day				
	olanzapine			amisulpride 400 mg/day				
Düring et al. (2019)	amisulpride	Prospective cohort study	56 (35/21)	Individualized dosing	NR	adults	schizophrenia patients	NR
Müller et al. (2006)	amisulpride	Prospective naturalistic study	99 (61/38)	400–1,200 mg/day	NR	adults	schizophrenia patients	amisulpride plasma level was higher in women
Kraal et al. (2017)	clozapine olanzapine	Cross-sectional study	79 (51/28)	Individualized dosing	63% White	adults	schizophrenia patients	NR
					25% Black			
					12% Other			
Lau et al. (2016)	clozapine	Retrospective cohort study	117 (67/50)	Individualized dosing	NR	NR	patients	NR
Hollingworth et al. (2018)	clozapine	Retrospective descriptive study	2,194 (1,470/724)	Individualized dosing	NR	NR	patients	NR
Pu et al. (2020)	olanzapine aripiprazole	Prospective cohort study	569 (283/286)	risperidone 3–6 mg/day olanzapine 10–25 mg/day aripiprazole 15–30 mg/day	East Asian	adults	schizophrenia patients	NR
	risperidone							
Belmonte et al. (2016)	Aripiprazole	Retrospective meta-analysis	157 (89/68)	10 mg single dose	NR	adults	healthy volunteers	AUC and Cmax were higher in women
Labelle et al. (2001)	risperidone	Prospective cohort study	330 (232/98)	6 mg/day	NR	adults	schizophrenia patients	NR
Cardiovascular Medications								
Essebag et al. (2007)	amiodarone	Prospective cohort study	973 (583/390)	Individualized dosing	NR	adults	AF patients	NR
Roten et al. (2009)	amiodarone	Retrospective chart review	264 (192/72)	Individualized dosing	NR	adults	AF patient	NR
Lehmann et al. (1996)	d,l-sotalol	Retrospective study	3,135 (2,336/799)	Individualized dosing	NR	Adults	patients	NR
Smiderle et al. (2014)	simvastatin atorvastatin	Prospective cohort study	495 (164/331)	Individualized dosing	European descent	adults	hypercholesterolemia patients	NR
Sadanaga et al. (2009)	enalapril	Prospective cohort study	199 (101/98)	Individualized dosing	NR	adults	HF patients	NR
Ishani et al. (2005)	enalapril	Retrospective study	6,436 (5,458/978)	10 mg bid	86% Caucasian	adults	HF patients	NR
					7% AA			
					2% Hispanic			
					6% other			
Wood (1995)	enalapril captopril lisinopril	Retrospective cohort study	1,013 (547/466)	Individualized dosing	NR	adults	HTN patients	NR
Fan et al. (2008)	captopril nifedipine	Prospective, randomized study	3,535 (1,209/2,326)	captopril 25–50 mg/day	East Asian	adults	HTN patients	NR
	HCTZ atenolol			Nifedipine 20–40 mg/day				
				HCTZ 12.5–25 mg/day atenolol 12.5–25 mg/day				
Os et al. (1994)	lisinopril	Prospective, randomized study	828 (424/404)	Individualized dosing	NR	adults	HTN patients	NR
	Nifedipine							
Coulter and Edwards (1987)	captopril enalapril	Retrospective postmarketing surveillance	unknown	Individualized dosing	NR	children and adults	HTN patients	NR
Abad-Santos et al. (2005)	amlodipine	Prospective, randomized study	36 (18/18)	10 mg single dose	Caucasian	adults	healthy volunteers	similar AUC and Cmax in both sexes
Analgesic Medications								
Sadhasivam et al. (2015)	morphine	Prospective cohort study	219 (105/114)	0.1–0.2 mg/kg, additional 0.05 mg/kg PRN.	Caucasian	children	children undergoing tonsillectomy	NR
Fillingim et al. (2005)	morphine	Prospective, randomized, double-blind study	100 (39/61)	0.08 mg/kg single dose	Predominately Caucasian	adults	healthy volunteers	NR
Bijur et al. (2008)	morphine	Retrospective meta-analysis	355 (144/211)	0.1 mg/kg single dose	predominately Latino and African American	adults	patients with acute pain	NR

Individualized dosing: participants used their original dosage and might adjust the dose according to their heathy condition during the study. The researchers did not assign dose for participants. *HCTZ, hydrochlorothiazide; AF, atrial fibrillation; HF, heart failure; HTN, hypertension; AA, african american; bid, twice daily; PRN, as needed; NR, not reported; AUC, area under the curve; Cmax, maximum serum concentration.

### 3.2 Psychotropic medications

#### 3.2.1 Lithium

Lithium is recommended as the first-line treatment for both acute mania and maintenance phase in bipolar disorder (Yatham et al., 2018). Recent evidence has also suggested the value of lithium in reducing suicidal rate in patients with bipolar or major depression disorder (Smith and Cipriani, 2017). Despite its significant clinical benefits, lithium has gradually become less widely utilized due to its narrow therapeutic index and requirement for frequent blood tests. Some common ADRs of lithium are tremor, polyuria, hypothyroidism, weight gain, and increased thirst. Other more severe ADRs such as bradycardia, sinus node dysfunction, and seizure might happen at a lower rate.

Sex differences were identified in lithium-related thyroid dysfunction, tremor, weight gain, and oedema. Özerdem et al. (2014) assessed sex differences in lithium associated thyroid dysfunction through a retrospective, naturalistic study. One hundred four men and 136 women taking lithium for bipolar disorder with thyroid-stimulating hormone (TSH) level available were included in the study. Using 0.3–3 µIU/mL as the normal range of TSH, the researchers found that significantly fewer female patients (55.9%) were within the normal range compared to male patients (71.2%) (*p* = 0.016). Notably, the difference in the proportion of normal TSH between male and female patients was not significant in the non-lithium treated group, which suggested that the observed sex differences in thyroid dysfunction is related to lithium treatment rather than the disease state. The vulnerability to thyroid dysfunction in lithium-treated women has also been observed by Chantal Henry in another retrospective study (Henry, 2002). By interviewing 22 male and 38 female patients about lithium ADRs, the researcher found that more female patients than male patients reported new diagnosis of hypothyroidism during the first year of lithium treatment (37% vs. 9%, *p* < 0.05). Weight gain was also shown to affect more female than male patients (47% vs. 18%, *p* < 0.05) in the same study whereas tremor was more pronounced in male than female patients (54% vs. 26%, *p* < 0.05). There is a more recent retrospective study investigating reasons for lithium discontinuation performed by Öhlund et al. (2018). The results showed that female patients were more likely to discontinue lithium due to weight gain (*p* < 0.01) and oedema (*p* < 0.01) compared to male patients. To conclude, current evidence suggested that lithium-associated thyroid dysfunction, weight gain, and oedema affect more female patients, while lithium-associated tremor affect more male patients in the treatment of bipolar disorder.

#### 3.2.2 Amisulpride

Amisulpride is an atypical antipsychotic with selective blockade of dopamine 2 and dopamine 3 receptors. It has been reported by multiple studies to be an effective and well-tolerated treatment for schizophrenia (Puech et al., 1998; Leucht et al., 2013). More recently, the clinical significance of amisulpride has been evaluated in combination therapies with other antipsychotics such as olanzapine in treatment-resistant schizophrenia (Schmidt-Kraepelin et al., 2022; Woo et al., 2022). On the safety prospective, amisulpride is associated with increased prolactin level, weight gain, hypotension, sexual dysfunction, and prolonged QT interval.

As one of the well-established adverse events of amisulpride, increased prolactin level was reported to be sex-biased by multiple studies (Düring et al., 2019; Hoekstra et al., 2021). In the BeSt InTro study, 93 men and 51 women with schizophrenia diagnosis were randomized to different antipsychotics including amisulpride (Johnsen et al., 2020). When comparing amisulpride induced ADRs between sexes, the researchers found that women had significantly higher mean prolactin level (1,869 mIU/L) then men (920 mIU/L) under amisulpride treatment (*p* < 0.001) (Hoekstra et al., 2021). Further evaluations showed that the serum level of amisulpride was higher in women than in men after adjusting for the daily dose (*p* = 0.019), which might explain the observed female-biased ADR. As a potential consequence of elevated prolactin level (Halbreich et al., 2003), sexual disturbance was also evaluated in this study. Using Udvalg for Kliniske Undersøgelser side effect score (UKU score) as the measurement for sexual disturbance, the researchers found that women had more sexual disturbance compared to men with marginal significance (*p* = 0.051). Notably, similar findings were observed in a separate study conducted by Düring et al., 2019. By following 35 men and 21 women with schizophrenia taking amisulpride monotherapy, the researchers found that prolactin level was higher in women (*p* < 0.01) compared to men after 6 weeks of amisulpride treatment. Women also reported higher sexual dysfunction load than men did (*p* < 0.01). In conclusion, amisulpride related prolactin elevation and sexual dysfunction are more common in women than in men in treating schizophrenia, even though the average daily dose is similar between two sexes.

#### 3.2.3 Clozapine and olanzapine

Clozapine and olanzapine are both atypical antipsychotics with similar molecular structures. Clozapine is known as one of the most effective antipsychotics and it is the gold standard for treatment resistant schizophrenia. However, studies have shown that the use of clozapine in schizophrenia is suboptimal (Warnez and Alessi-Severini, 2014), which might involve several reasons including a range of serious adverse events of this medication. For instance, clozapine is associated with myocarditis, cardiomyopathy, and neutropenia, all of which can be life-threatening. Recently, the use of olanzapine in treatment resistant schizophrenia has been widely discussed, as several studies have shown that olanzapine is non-inferior to clozapine in terms of safety and efficacy in hard-to-treat schizophrenia (Tollefson et al., 2001; Bitter et al., 2004; Naber et al., 2005). In terms of common adverse events, both clozapine and olanzapine are recognized as being high risk for weight gain, hyperglycemia, and dyslipidemia (Rummel-Kluge et al., 2010; Kraal et al., 2017).

Even though clozapine and olanzapine have similar profiles in metabolic ADRs, the impact of sex on some of those ADRs were observed to be different between the two medications. In the BeSt InTro study (Hoekstra et al., 2021), sex differences in BMI increase was evaluated in patients randomized to olanzapine group. BMI increase was found to be more pronounced in men (1.48 kg/m^2^) than in women (0.24 kg/m^2^) (*p* < 0.001). Interestingly, the direction of sex difference in treatment-related weight gain was shown to be opposite in patients taking clozapine. In a retrospective study conducted by Lau et al., 2016, 67 men and 50 women attending the outpatient clozapine clinic were recruited and their weight change from 3 months to 12 months after clozapine initiation was calculated. The percentage weight change (weight change divided by the 3-month weight) was found to be significantly higher in women (+5.5%) than in men (+1.3%) (*p* = 0.01). To analyze sex differences in more serious ADRs of clozapine, Hollingworth et al. reviewed all reported clozapine related neutropenia, myocarditis, and cardiomyopathy cases in Australia monitoring database from 1993 to 2014 (Hollingworth et al., 2018). Sex differences were observed with neutropenia happening more in women (OR 1.45, CI 1.28–1.67), while cardiomyopathy (OR 2.53, CI 1.9–3.37) and myocarditis (OR 1.58, CI 1.34–1.87) happened more in men. These findings suggest sex as an important factor in clozapine and olanzapine related weight gain as well as in more serious adverse events of clozapine.

#### 3.2.4 Aripiprazole

Aripiprazole is an atypical antipsychotic with numerous FDA approved indications including schizophrenia, bipolar I disorder, autistic disorder, Tourette’s syndrome, and major depressive disorder. Because of its unique receptor binding profile, aripiprazole has different mechanism of actions from other antipsychotics and is sometimes referred as a third-generation antipsychotic (Freudenreich and Freudenreich, 2020). In addition to its confirmed efficacy in various disease areas, aripiprazole has also been shown to induce less adverse events compared with other antipsychotics (Leucht et al., 2013). Some common ADRs of aripiprazole are weight gain, nausea, vomiting, tremor, and fatigue. More serious ADRs such as prolonged QT interval, myocardial infarction, and neutropenia have been observed at a lower rate.

Among aripiprazole-related ADRs, weight gain and some cardiovascular ADRs were shown to impact men and women differently. In a study evaluating sex differences in pharmacokinetics and ADRs of aripiprazole, 89 men and 68 women from multiple aripiprazole bioequivalence clinical trials were recruited (Belmonte et al., 2016). PK parameters were calculated, and physical assessments were performed several times before and after a single dose of 10 mg aripiprazole. The study found that AUC and C_max_ of aripiprazole were significantly higher in women (*p* < 0.05), which indicated a higher aripiprazole exposure in women even under the same dose. In concordance with the observed difference in PK parameters, the blood pressure lowering effects of aripiprazole were found to be more pronounced in women at all measured times (*p* < 0.01). At 8 h after the dose, the mean systolic blood pressure in women was 105 mmHg versus 116 mmHg in men (*p* < 0.001). In addition, women were found to have higher heart rate and larger QTc interval compared to men at multiple measured times (*p* < 0.001). As a well-established ADR of aripiprazole, weight gain has also been shown to impact women and men differently. In the BeSt InTro study (Hoekstra et al., 2021), men were observed to have higher BMI increase compared to women after 52 weeks of aripiprazole use (0.64 kg/m^2^ vs. −0.04 kg/m^2^, *p* = 0.016). In conclusion, sex differences have been observed in multiple aripiprazole related ADRs including weight gain, blood pressure reduction, increased heart rate and QTc. Since some of the conclusions were based on a single dose of aripiprazole, further investigation is warranted to explore the sex difference in long-term aripiprazole use.

#### 3.2.5 Risperidone

Risperidone is a second-generation antipsychotic with serotonin 5-hydroxytryptamine receptor 2 (5-HT_2_) blocking activities at low doses and dopamine D_2_ receptor blocking activities at higher doses (Megens et al., 1994). Risperidone is proven to mitigate both positive and negative symptoms of schizophrenia, with less concern about dyskinesia which is a prevalent ADR of most antipsychotics (Labelle et al., 2001). Some common ADRs of risperidone are rash, weight gain, hyperprolactinemia, parkinsonism, and fatigue.

Sex differences in risperidone-associated rash, weight gain, parkinsonism, and dystonia have been evaluated. In a randomized study, 100 men and 90 women taking daily risperidone were followed up for 1 year to assess drug-related ADRs (Pu et al., 2020). At the end of the follow-up period, more female patients reported rash related to risperidone than male patients (*p* = 0.03). In another *post hoc* analysis on an open-label study, ADRs in 232 men and 98 women taking risperidone were analyzed for differences between sexes (Labelle et al., 2001). Weight gain was found to happen more in men compared to women with marginal significance (*p* = 0.085). No sex difference was identified for parkinsonism (*p* = 0.889) or dystonia (*p* = 0.512). To conclude, risperidone-related rash is more prevalent in women, whereas no significant sex difference was found in weight gain, parkinsonism, or dystonia related to risperidone treatment in schizophrenia.

### 3.3 Cardiovascular medications

#### 3.3.1 Amiodarone

Amiodarone is a class III antiarrhythmic drug which is highly effective and widely used in both supraventricular and ventricular arrhythmias (Connolly, 1999). However, amiodarone is also well-known for its potential ADRs on different organs such as thyroid, heart, lung, liver, and eyes. A previous study showed that the prevalence of amiodarone-related ADRs is 15% in the first year, and may increase to 50% in long term use, which would ultimately lead to medication discontinuation in 20%–50% of the patients (van Erven and Schalij, 2010). Some common ADRs of amiodarone are thyroid dysfunction, photosensitivity, and visual disturbance. Amiodarone can also cause more serious adverse events such as bradyarrhythmia, sinus arrest, and hepatotoxicity.

In a prospective cohort study (Essebag et al., 2007), Essebag et al. enrolled 583 men and 390 women with new onset atrial fibrillation (AF) and followed the participants for up to 30 months for amiodarone related ADRs. The researchers found that amiodarone use was associated with increased risk of pacemaker insertion only in women but not in men (HR: 4.69, 95% CI: 1.99–11.05, vs. HR: 1.05, 95% CI: 0.42–2.58, *p* = 0.02). This significant difference remained after adjusting for daily dose, weight, and the use of other antiarrhythmic medications. In another retrospective study (Roten et al., 2009), Roten et al. reviewed amiodarone associated ADRs in 192 men and 72 women who were referred to clinic for AF management. Their analysis showed that women overall experienced more amiodarone-related ADRs than men (56% vs. 36%, *p* = 0.046), and there were significant sex differences in the occurrence of phototoxicity under amiodarone treatment (21% in women vs. 8% in men, *p* = 0.047). The results above suggest that closer monitoring is needed in female population taking amiodarone since they are more likely to experience ADRs such as bradyarrhythmia requiring pacemaker insertion and phototoxicity.

#### 3.3.2 Sotalol

Sotalol is a class III antiarrhythmic agent which is approved for treatment of AF and ventricular arrhythmia. Its efficacy in reducing death and preventing recurrence of arrhythmia has been proven to be superior to other antiarrhythmic drugs (Mason, 1993). However, along with its high efficacy, sotalol can induce some lethal ADRs such as Torsades de pointes (TdP), which may lead to sudden cardiac death. To unveil whether sex is a risk factor for sotalol induced TdP, Lehmann et al. assessed the prevalence of TdP development under sotalol treatment in 3,135 adult patients and compared the results between sexes (Lehmann et al., 1996). TdP was observed in 44 of 2,336 men (1.9%) and in 33 of 799 women (4.1%), and the difference was statistically significant (*p* < 0.001). Further logistic regression also suggested female sex as a significant risk factor in TdP development (*p* < 0.0001), even after adjusting for sotalol dose. Since TdP is such a lethal ADR, the results above emphasize the need for closer monitoring of cardiac function in female patients taking sotalol.

#### 3.3.3 Simvastatin and atorvastatin

Despite the recent advancement in the treatment options for hyperlipidemia and in the prevention of coronary artery disease, statins remain the first line therapy due to their high efficacy, low cost, and relatively safe profile. The pharmacological effects of statins have been proven in lowering the low density lipoprotein cholesterol (LDL-C) by 20%–50%, as well as lowering triglyceride by 10%–20% (Taylor et al., 2013). In terms of safety, statins are well tolerated by the vast majority of patients, but they can still cause some ADRs such as myalgias, urinary tract infection, and increased liver enzymes, which can all lead to treatment interruption or discontinuation. Sex differences in the ADRs of two commonly used statins, simvastatin and atorvastatin, have been evaluated in a prospective cohort study (Smiderle et al., 2014). A total of 164 men and 331 women on simvastatin or atorvastatin treatment participated in the study, and they were evaluated every 3 months for statin related ADRs. The researchers observed higher occurrence of myalgia in women than in men (25.9% vs. 20.3%, *p* = 0.002), while more creatinine phosphokinase (CPK) increase and/or elevated liver enzymes were observed in men than in women (11.1% vs. 7.6%, *p* = 0.017) under simvastatin or atorvastatin treatment. These results request more attention on the role of sex in statin associated ADRs, and further studies are warranted to explore the potential mechanism of the observed sex differences.

#### 3.3.4 Enalapril, lisinopril, and captopril

Angiotensin converting enzyme (ACE) inhibitors are effective antihypertensives working through inhibition of renin-angiotensin system. ACE inhibitors are recommended by multiple guidelines as first-line treatment for hypertension (Williams and Mancia, 2018), and their use has been expanded to other disease areas such as acute myocardial infarction, heart failure, and kidney diseases. While most patients tolerate ACE inhibitors well, some patients can still experience hypotension, dizziness, dry cough, and other more serious ADRs such as angioedema and renal impairment during the treatment.

Evidence of sex differences in ACE inhibitor induced ADRs was found in lisinopril, enalapril, and captopril. Interestingly, most of the sex difference analysis has been focused on ACE inhibitor induced bronchospasm and cough. In a retrospective study (Wood, 1995), the prevalence of new onset bronchospasm and cough was assessed in 1,013 patients taking captopril, lisinopril, or enalapril. Women were found to experience more bronchospasm (58% vs. 42%) and cough (59% vs. 41%) reactions compared to men; however, the difference was not statistically significant. Notably, patients under the three different treatments were not separated when the prevalence was reported, which means that the rate of bronchospasm and cough in each individual medication group was unknown. In another randomized, double-blind clinical trial investigating sex differences in efficacy and safety of antihypertensives, 3,535 hypertensive patients (1,209 men and 2,326 women) were recruited and followed during 8 weeks of treatment (Fan et al., 2008). In patients randomized to captopril group, the prevalence of cough was found to be significantly higher in women than in men (14.3% vs. 8.4%, *p* = 0.005). This female-biased ACE inhibitor induced cough was also observed in lisinopril by Os et al. in a randomized, double-blind clinical trial (Os et al., 1994). In this study, 206 men and 206 women were randomized to lisinopril group, and cough was found to happen three times more often in women than in men (12.6% vs. 4.4%, *p* = 0.0027). Overall, although some non-significant findings exist, more evidence suggests an increased risk of ACE inhibitor induced cough in women.

#### 3.3.5 Amlodipine and nifedipine

Both amlodipine and nifedipine are dihydropyridine calcium channel blockers (CCBs) which are widely used for treating hypertension, stable and variant angina. Although structurally similar, amlodipine differs from nifedipine and other dihydropyridine CCBs by its long half-life, enabling once daily dosing (Haria and WagstaffAmlodipine, 1995). In terms of ADRs, both amlodipine and nifedipine are observed to cause hypotension, palpations, edema, and flushing with slightly different occurrence rate.

Sex difference studies are available for amlodipine-related neurological ADRs and nifedipine-related cough and edema. Abad Santos et al. conducted a bioequivalent study in 36 healthy volunteers (18 men and 18 women) to study sex differences in amlodipine induced ADRs as their secondary objective (Abad-Santos et al., 2005). All subjects received a single 10 mg dose of each amlodipine formulation with a 14-day washout period. After statistical analysis, the researchers did not find any significant difference between men and women in amlodipine related headache (44% vs. 28%), dizziness (11% vs. 28%), or tiredness (17% vs. 6%). Sex difference in nifedipine-related edema was studied in a prospective study by Fan et al. (Fan et al., 2008). A total of 327 men and 620 women were randomized to nifedipine sustained release (SR) group and were followed up for 8 weeks to evaluate drug related ADRs. Women were found to be more susceptible to ADRs related to nifedipine SR than their men counterpart (15.8% vs. 9.8%, *p* = 0.017), with intolerable edema being the main type of ADR observed. In another study assessing the role of sex in nifedipine associated cough, 218 men and 198 women were randomized to nifedipine group and were followed up for 10 weeks (Os et al., 1994). No sex difference was identified by this study in nifedipine related cough (men 3% vs. women 2.8%). To conclude, women were found to experience more intolerable edema from nifedipine SR, while no sex difference was found in nifedipine associated cough or amlodipine associated headache, dizziness, or tiredness.

#### 3.3.6 Atenolol

Atenolol is one of the drugs classified as beta-blocker, and it is used to treat several conditions such as hypertension, cardiac dysrhythmia, angina pectoris, etc. Recently, the effectiveness of atenolol has been assessed in other disease areas including anxiety (Armstrong and Kapolowicz, 2020). In terms of its safety profile, most patients tolerate atenolol well. Bradyarrhythmia, hypotension, dizziness, and fatigue are the most common ADRs observed with atenolol treatment. There is one study evaluating sex difference in ADRs related to atenolol in treating hypertension. After following 191 men and 403 women on atenolol therapy for 8 weeks, the researchers found that fatigue and bradycardia were most common ADRs during treatment period, and there was no sex difference in the occurrence rate of those ADRs (men 15.8% vs. women 11.6%, *p* = 0.497) (Fan et al., 2008).

### 3.4 Analgesic medications

#### 3.4.1 Morphine

Opioids are widely used in the management of moderate to severe pain. As one of the potent opioid analgesia, morphine is recommended for pain management in various disease types such as cancer, acute pulmonary edema, and myocardial infarction (Task Force on the management of ST-segment elevation acute myocardial infarction of the European Society of Cardiology ESC, 2012; Wiffen et al., 2016). However, the use of morphine has been cautioned due to a wide range of ADRs including pruritus, nausea, vomiting, dizziness, urinary retention, and more seriously, drug dependence, respiratory depression, and cardiac arrest.

Sex differences have been investigated in multiple morphine induced ADRs such as gastrointestinal ADRs and respiratory depression. In a prospective observational study undertaken by Sadhasivam et al. (Sadhasivam et al., 2015), 219 children undergoing tonsillectomy or adenotonsillectomy (T/TC) surgery were recruited and the efficacy and safety of morphine were compared between boys and girls. No sex difference was observed in respiratory depression (10% in boys vs. 7% in girls, *p* = 0.81), postoperative nausea and vomiting (6% in boys vs. 9% in girls, *p* = 0.2), and pruritus (41% in boys vs. 33% in girls, *p* = 0.54). Likewise, sex differences in morphine related ADRs were also assessed by Fillingim et al. in healthy adult women (n = 61) and men (n = 39) (Fillingim et al., 2005). All subjects in the study were intravenously administered 0.08 mg/kg single dose of morphine, after which the incidence of pruritus, nausea, and emesis were assessed. Similar to the previously described study, no evidence of sex difference was found in pruritus (8% in men vs. 10% in women). However, the prevalence of nausea and emesis were found to be significantly higher in women than in men (nausea 35% vs. 3%, emesis 18% vs. 0, *p* < 0.005). The results from the two studies above indicates that the role of sex in morphine related nausea and vomiting might be different in different disease states and/or age groups.

## 4 Discussion

**FIGURE 2:**
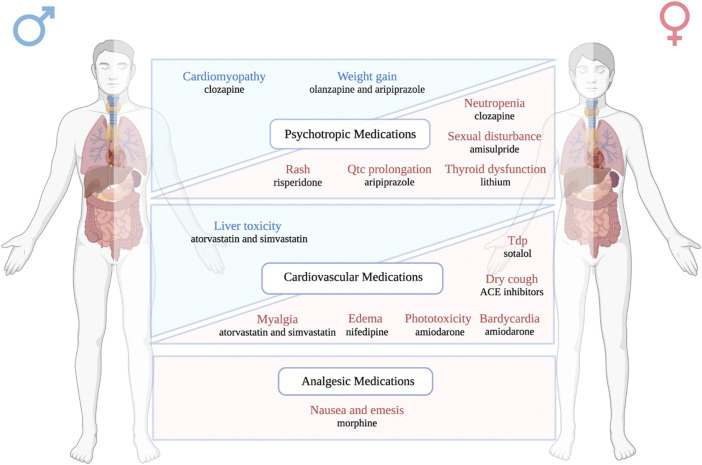
A schematic figure listing the main ADRs showing sex differences in occurrence rate. The adverse drug reactions highlighted in blue are male-biased ADRs, while the adverse drug reactions highlighted in red are female-biased ADRs. Drugs and their associated sex biased ADRs are classified into three different therapeutic categories: psychotropic medications, cardiovascular medications, and analgesic medications.

Despite the careful premarketing evaluation and postmarketing surveillance, adverse drug reactions remain a global public health issue leading to morbidity, mortality, and huge financial loss. In the United States, severe ADRs have been estimated to occur more than 2 million times in hospitalized patients every year, which ultimately result in 100,000 deaths (Giacomini et al., 2007). The financial burden caused by ADRs has been calculated to be equivalent to 16% of total healthcare expenditures in the US in 2016 (Watanabe et al., 2018). Although some recent efforts have been invested into ADR prediction (Lounkine et al., 2012; Mohsen et al., 2021; Zhang et al., 2021), it remains challenging to identify patients with high risk to develop certain ADRs clinically, which might be due to lack of data, limited sample size of ADR studies, and the complex nature of ADR generation, etc. As a ready-to-use clinical character, sex has recently been shown to be an influencer in the risk of ADR development (Tharpe, 2011; Nakagawa and Kajiwara, 2015). Here, we systematically reviewed the role of sex in the risk of ADRs caused by commonly used psychotropic, cardiovascular, and analgesic medications. Our findings suggested that several common and/or severe ADRs have difference prevalence in men versus in women as shown in Figure 2.

Quantitively, we included studies evaluating sex differences in ADR occurrence for 6 psychotropic medications, with 18 drug-specific ADRs showing sex differences, 15 drug-specific ADRs showing no sex difference; 10 cardiovascular medications, with 8 drug-specific ADRs showing sex differences, 4 drug-specific ADRs showing no sex difference; 1 analgesic medication with 3 drug-related ADRs showing no sex difference. The 17 drugs discussed in this review cover a variety of disease areas such as bipolar disorder, schizophrenia, arrhythmia, hypertension, hyperlipidemia, pain, etc. Notably, as an important class of psychotropic medication, the antidepressant medications in our searching list did not result in any study showing sex differences in ADR, which implies that more sex-awareness is needed for this particular drug class. A complete list of the sex difference findings in ADR can be found in Table 2.

**TABLE 2:** Summary of findings of sex difference research.

Drug	Consistent findings			Conflicting findings
	Male-biased	Female-biased	No sex difference	
Psychotropic medications				
Lithium	Tremor (Henry, 2002)	Edema (Öhlund et al., 2018)	Acne (Henry, 2002)	—
		Weight gain (Henry, 2002; Öhlund et al., 2018)	Polyuria (Henry, 2002)	
		Thyroid dysfunction (Henry, 2002; Özerdem et al., 2014)		
Amisulpride	—	Increased prolactin levels (Düring et al., 2019; Hoekstra et al., 2021)	Extrapyramidal symptoms (Müller et al., 2006; Hoekstra et al., 2021)	Sexual dysfunction (female-biased (Düring et al., 2019) vs. no sex difference (Müller et al., 2006; Hoekstra et al., 2021))
			Agitation (Müller et al., 2006)	
			Sedation (Müller et al., 2006)	
			Blurred vision (Müller et al., 2006)	
			Metabolic ADRs (Hoekstra et al., 2021)	
			Hypersalivation (Müller et al., 2006)	
Clozapine	Cardiomyopathy and myocarditis (Hollingworth et al., 2018)	Weight gain (Lau et al., 2016)	—	—
		Neutropenia (Hollingworth et al., 2018)		
Olanzapine	Increased BMI (Hoekstra et al., 2021)	Dermatological symptoms (Pu et al., 2020)	Dystonia, rigidity, hypo/hyperkinesia, tremor, seizure (Hoekstra et al., 2021)	—
	Increased glucose level (Hoekstra et al., 2021)	Higher prolactin level (Hoekstra et al., 2021)	Sexual dysfunction (Hoekstra et al., 2021)	
		Autonomic ADRs (Pu et al., 2020)		
Aripiprazole	BMI increase (Hoekstra et al., 2021)	BP lowering, higher HR, prolonged QTc interval (Belmonte et al., 2016)	Dystonia, rigidity, hypo/hyperkinesia, tremor, seizure (Hoekstra et al., 2021)	—
		Nausea and vomiting (Belmonte et al., 2016)	Increased glucose level (Hoekstra et al., 2021)	
		Psychotic ADRs (Pu et al., 2020)	Sexual dysfunction (Hoekstra et al., 2021)	
Risperidone	—	Rashes (Pu et al., 2020)	Weight gain (Labelle et al., 2001)	—
			Parkinsonism, dystonia (Labelle et al., 2001)	
Cardiovascular medications				
Amiodarone	—	Phototoxicity (Roten et al., 2009)	—	—
		Bradyarrhythmia requiring pacemaker insertion (Essebag et al., 2007)		
d,l-sotalol	—	Torsade de pointes (TdP) (Lehmann et al., 1996)	—	—
Simvastatin/atorvastatin	Abnormal liver function (Smiderle et al., 2014)	Myalgia (Smiderle et al., 2014)	—	—
	Increased CPK levels (Smiderle et al., 2014)			
Enalapril	Anemia (Ishani et al., 2005)	—	—	Cough (female-biased (Coulter and Edwards, 1987) vs. no sex difference (Wood, 1995; Sadanaga et al., 2009))
Captopril	—	—	—	Cough (female-biased (Coulter and Edwards, 1987; Fan et al., 2008) vs. no sex difference (Wood, 1995))
Lisinopril	—	—	—	Cough (female-biased (Os et al., 1994) vs. no sex difference (Wood, 1995))
Amlodipine	—	—	Headache, dizziness, and tiredness (Abad-Santos et al., 2005)	—
Nifedipine	—	Intolerable edema (Fan et al., 2008)	Cough (Os et al., 1994)	—
Atenolol	—	—	Bradycardia (Fan et al., 2008)	—
			Fatigue (Fan et al., 2008)	
Analgesic medications				
Morphine	—	—	Pruritus (Fillingim et al., 2005; Sadhasivam et al., 2015)	Nausea and vomiting (female-biased (Fillingim et al., 2005) vs. no sex difference (Bijur et al., 2008; Sadhasivam et al., 2015))
			Dizziness (Fillingim et al., 2005)	
			Respiratory depression (Sadhasivam et al., 2015)	

* BMI, body mass index; BP, blood pressure; HR, heart rate; CPK, creatinine phosphokinase.

Intriguingly, we identified some well-established ADRs which were shown to exert sex difference patterns by multiple studies. For instance, lithium was found to cause more thyroid dysfunction in women than in men (Henry, 2002; Özerdem et al., 2014), and amisulpride was shown to increase prolactin level more in women than in men (Düring et al., 2019; Hoekstra et al., 2021). In addition to the consistent findings on sex biased ADRs, sex difference research in serious ADRs is also worth mentioning. As a rare but life threatening ADR of clozapine, neutropenia was found to happen more in women than in men in a retrospective study (Hollingworth et al., 2018), suggesting that more surveillance is needed for women with long-term clozapine use. Similarly, after reviewing the ADRs in patients treated with sotalol for arrhythmia, researchers found that more women developed TdP, a fatal ADR of sotalol, than men (Lehmann et al., 1996). These clinically observed ADRs should serve as stimulants for both consideration of sex in drug selection and ADR monitoring, as well as future studies to explore the underlying mechanism behind the observed sex differences.

In addition to the findings showing consistent sex differences in certain ADRs, conflicting results also exist, which makes it difficult to draw a certain conclusion. For instance, morphine-associated nausea and vomiting was concluded as female-biased by Fillingim et al. (Fillingim et al., 2005), whereas no sex difference was observed in the same ADR in another study (Sadhasivam et al., 2015). After carefully reviewed the two studies, we found that the former study recruited healthy adult volunteers, while the latter one recruited children undergoing tonsillectomy or adenotonsillectomy (T/TC) surgery. The distinct target populations made it difficult to compare the results between the two studies, since both age and disease state can impact the risk of drug ADRs (Lavan and Gallagher, 2016). Similarly, other discrepancies in the study design (dosing regimen, follow-up time, definition of certain ADR, ethnicity group of the participants, etc.) also introduce complexities when results were compared between studies. Therefore, we suggest that more thorough study design and more robust methods such as meta-analyses are needed to better understand sex differences in the risk of ADR generation.

For all the studies that are included in this review, we searched the article for potential mechanisms that may explain the observed sex differences. Surprisingly, only five out of the twenty-six studies discussed the putative underlying mechanisms, all of which are related to differences in the serum concentration of the medication between men and women. However, in-depth discussion on the reason of the differences in PK profile between sexes is missing in those studies. In fact, there are recent publications summarizing how sex might impact PK and drug response. It is believed that the intracellular and extracellular water volumes, amount of fat mass, expression of drug metabolizing enzymes and transporters, and glomerular filtration might be different between men and women, which can impact every aspect of absorption, distribution, metabolism, and elimination of a medication (Gandhi et al., 2004; Soldin et al., 2011; Yang et al., 2012). More broadly speaking, other factors such as genetics, hormone, immune system, microorganisms, and environment could also contribute to sex differences in drug efficacy and safety by impacting PK and/or pharmacodynamic of medications (Arnold, 2017; Weersma et al., 2020; Cheng et al., 2022; Huang et al., 2023). Therefore, we suggest that future studies need to consider a wider range of potential mechanisms to better understand the observed sex differences in drug ADRs.

Our study has some limitations. Although the 1,819 drug list used for web scraping covers the majority of the most prescribed cardiovascular, psychotropic, and analgesic medications (Fuentes et al., 2018) (22/24 top 100 cardiovascular medications, 15/15 top 100 psychotropic medications, 9/9 top 100 analgesic medications are in drug list), we are missing two commonly prescribed cardiovascular medications which are furosemide and aspirin. We manually searched evidence of sex differences in ADR related to the above two medications using the same criteria as listed in Figure 1, which resulted in one study showing sex differences in reported bleeding events related to aspirin (Rydberg et al., 2014). This retrospective study found that women were at a lower risk of aspirin related bleeding compared to men (RR 0.8, 95% CI 0.66-1.96). Since we did not use an exhaustive list of cardiovascular, psychotropic, and analgesic medications, one limitation of our study is that we might miss evidence of sex differences in ADRs related to some less commonly used medications under the three categories above. Second, the distinct quality and study design (dosage, route of administration, target population, etc.) of the included studies introduce complexities when comparing the results among the studies. For instance, we found that differences in the risk of bias of the included studies may contribute to conflicting results. Using Risk Of Bias In Non-randomized Studies - of Intervention (ROBINS-I) as the tool (Sterne et al., 2016), we found that the study conducted by Müller et al. (2006) has a moderate risk of bias due to confounding because of its naturalistic study design and the different dosage of amisulpride used by participants. In comparison, the study conducted by Hoekstra et al. (2021) has a low risk of bias due to confounding since the patients received the same dose of amisulpride. This difference in risk of bias may be able to explain the conflicting finding of the two studies on sex differences in sexual disfunction related to amisulpride. Therefore, we suggest that the results of this review should be carefully interpreted with the quality and design of the original study. Third, our search results are exclusively generated from PubMed search. A more comprehensive list of relevant studies might be achieved by including other databases such as Cochrane Library and Web of Science.

Overall, sex differences in ADRs have been studied and identified in a handful of psychotropic, cardiovascular, and analgesic medications. However, to better understand the underlying mechanism of the observed sex differences in ADRs, further studies with more comprehensive study design are warranted. Some key factors to consider are clearly documented ADRs in each sex group, collection of PK data, pharmacogenomic data, measurement of microorganism, document of environmental exposure, etc. It is of great clinical significance to understand how sex can impact the risk of ADRs so that more personalized approaches could be applied to minimize the burden caused by ADRs.

## Data Availability

The original contributions presented in the study are included in the article/Supplementary Material, further inquiries can be directed to the corresponding author.
