# Supplementary material for: A systematic review on sex differences in adverse drug reactions related to psychotropic, cardiovascular, and analgesic medications
Source: Front Pharmacol. 2023 May 2;14:1096366. doi: 10.3389/fphar.2023.1096366 (PMC10185891; doi:10.3389/fphar.2023.1096366)
Supplement: Supplementary file 1 [file Table1.DOCX]

**Supplemental Table 1. Summary of Searching Strategy**

| Items | Specification |
| --- | --- |
| Date of search | March 2022 |
| Date of publication | No restriction |
| Database | PubMed |
| Search terms | 1819 drugs of interest AND  sex difference OR sex differences OR gender difference OR gender differences AND  toxicity OR toxicities OR adverse OR side effect OR side effects NO  mice OR mouse OR rat OR rats |
